# Bibliometric analysis of scientific publications on “sustainable development goals” with emphasis on “good health and well-being” goal (2015–2019)

**DOI:** 10.1186/s12992-020-00602-2

**Published:** 2020-07-28

**Authors:** Waleed M. Sweileh

**Affiliations:** Department of Physiology, Pharmacology/Toxicology, Division of Biomedical Sciences, College of Medicine and Health Sciences, An-Najah National University, Nablus, Palestine

**Keywords:** Sustainable development goals, Bibliometric analysis, Scopus

## Abstract

**Background:**

Global progress in the United Nations’ Sustainable Development Goals (SDGs) requires significant national and international research efforts and collaboration. The current study aimed to provide policymakers, academics, and researchers with a snapshot of global SDGs-related research activity.

**Method:**

This was a cross-sectional descriptive bibliometric study. SciVerse Scopus was used to retrieve SDGs-related research publications for the period from 2015 to 2019.

**Results:**

In total, 18,696 documents were found. The *Sustainability* journal ranked first (*n* = 1008; 5.4%) in the number of SDGs-related publications. The World Health Organization was the most active institution in publishing SDGs-related documents (*n* = 581; 1.3%). Most of the retrieved documents belonged to SDG 17 (partnership) followed by SDG 13 (climate action), and SDG 12 (responsible consumption and production), while SDG 7 (affordable and clean energy) had the least number of publications. The European region (*n* = 9756; 52.2%) had the highest research contribution while the Eastern Mediterranean region (*n* = 1052; 5.6%) had the least contribution. After exclusion of SDG 17, the SDG 3 (good health and well-being) was the top researched SDG for the African region, the Eastern Mediterranean regions, and the South-Eastern Asian region. For the region of the Americas, European region, and the Western Pacific region, the SDG 13 (climate action) was the most researched. The SDG 7 (affordable and clean energy) was the least researched in the African region, the region of the Americas, the European region, and the South-East Asian region. In the Eastern Mediterranean region, SDG 10 (reduced inequality) was the least researched while in the Western Pacific region, SDG 5 (gender inequality) was the least researched. The most researched targets of SDG 3 were targets 7 (sexual and reproductive health services) and 8 (universal health coverage) while the least researched targets were 5 (substance use disorders) and 9 (death from hazardous materials). International research collaboration within SDG 3 between high- and low-income countries was inadequate.

**Conclusion:**

The analysis presented in the current study are useful for researchers, institutes, governments, funding agencies, and policy-makers. Countries in Africa, the Middle East, and South-East Asia need to increase their funding and research collaboration in the field of SDGs.

## Background

The United Nations’ (UN) sustainable development goals (SDGs), adopted on September 2015, are a universal political agenda that call for a collective action to achieve peace, prosperity, and well-being for all by 2030. The 17 goals and 169 targets of the SDGs were developed to succeed the eight Millennium Development Goals (MDGs) which ended in 2015 [1]. The transition from eight MDGs to 17 SDGs marked a shift from vertical to horizontal approach in addressing global issues [2]. Achieving cross-sectional progress in the ambitious 2030 agenda requires the involvement of policymakers at all levels and in all sectors [3]. The list of SDGs includes ending poverty, ending hunger, encouraging good health and well-being, providing quality education, promoting gender equality, providing clean water and sanitation, promoting affordable and clean energy, providing decent work and economic growth, addressing industry, innovation and infrastructure, reducing inequalities, developing sustainable cities and communities, encouraging responsible consumption and production, taking action on climate change, promoting life below water, promoting life on land, working towards peace, justice and strong institutions, and creating partnerships to achieve SDG goals [3].

The third SDG of good health and well-being is currently of special interest given the global health threats imposed by serious pandemics including the recent COVID-19 which might interrupt the national and international progress to achieve the 2030 goals [4, 5]. Global warming has also created a serious threat to human lives by accelerating the spread of certain infectious diseases, particularly mosquito-borne diseases which threaten millions of people around the world [6]. Furthermore, conflicts and fragility in various world regions including Africa and the Eastern Mediterranean regions created massive waves of refugees and migrants with minimum or poor health services [7, 8]. Poverty, poor sanitation, lack of access to essential medicines and health services have augmented the disease burden on most vulnerable categories particularly women, children, and elderly [9]. The global health challenges imposed by emerging pandemics, conflicts, climate change, and poor economic growth affect the plans to achieve the 17 SDGs particularly the third one focusing on good health and well-being.

Researchers and academic staff in universities are key players in promoting SDGs [10, 11]. Contribution of scientists in different disciplines enlightens policy-makers and politicians on certain goals that need to be addressed in the future as well as on the extent of success or failure in achieving a certain goal. The SDGs are based on the presence of serious global problems that need to be addressed and solved through research and innovation [12]. Researchers are in a position to provide genuine solutions and inventions to various national and international problems facing the pathway to achieving the 2030 agenda [10]. Local and international authorities need to adopt research results and recommendations made by academic staff and put them into a practical agenda to strengthen partnership with all stakeholders and to accelerate the practical steps toward achieving SDGs.

To shed light on the contribution of academics and researchers to SDGs-related literature, the current study was performed aiming to assess research volume and research trends of scientific publications on SDGs over the past 5 years (2015–2019). The analysis was based on traditional bibliometric methodology [13], which has been extensively used to analyze and assess a wide range of medical and scientific topics [14–16]. The current study will shed light on most and least-researched goals and targets. The third SDG will be given special consideration and analysis.

## Methods

### Search tool

The current study was a cross-sectional descriptive analysis of scientific literature on “sustainable development goals” retrieved from Scopus database. Scopus, an online database with approximately 23,000 available journals across all fields of research, was used [17]. Scopus is commonly used in bibliometric studies and is considered suitable for this purpose because it includes a larger number of indexed journals than Web of Science [18]. The current study was carried out on July 10th, 2020, and all data analysis, including citation analysis, was carried out on the same day.

### SDGs-related research

Research that supports national or global implementation and development to achieve SDG targets can be classified into two types: (1) those that do not mention the phrase “sustainable development goals” and (2) and those that explicitly mention the phrase “sustainable development goals”. The second type of research is directly related or linked to policies, implementation, barriers, and other aspects of the SDGs [19]. In the current study, we focused on the second type of research in which the phrase “sustainable development goal” was mentioned in the paper (title, abstract, introduction, methodology or discussion). These fields can be searched in any article using the function “ALL” in Scopus search engine. Of course, this type of research does not represent all research on SDGs. However, it will relatively reflect the contribution of various countries, world regions, journals, research themes and shed light on other indicators. Furthermore, the results of the analysis of this type of research can be used to compare the extent of research on various SDG goals among world regions with an acceptable level of accuracy.

### Search strategy

1. The search strategy was explained in Additional file 1. Documents with the phrase “sustainable development goal*” mentioned in any place in the paper were retrieved from Scopus for the study period from 2015 to 2019. The documents were limited to journal articles but no language restriction was imposed. The author believed that using this approach will retrieve the maximum potential number of documents of the second type in which the author(s) tried to link their study with DSGs. A very recently published article on SDGs used the title-abstract-key methodology to retrieve SDGs-relevant articles and retrieved approximately 4500 documents [19]. Using the methodology adopted in the current study, the number of SDG-related documents was at least three times larger than the number obtained from the title-abstract-key methodology. The 18,969 scientific publications obtained in the current study using the “ALL” function were considered to be SDGs-related publications and used for further research.
2. The search strategy for the number of SDGs-related documents contributed by each world region was calculated based on the methodology stated above and the list of all countries located in each world region. The world regions considered in the current study were those adopted by the WHO: African region, the region of the Americas, the European region, the South-East Asian region, the Eastern Mediterranean region, and the Western Pacific region.
3. The search strategy for each SDG goal was entirely based on the search queries for each SDG goal posted by “AURORA UNIVERSITIES NETWORK” [20], which is an initiative started from the Aurora Universities Network in 2017. For each goal and each target a search query is available and can be directly inserted into Scopus advance search system [21]. The search queries for the 17 SDG goals using the keywords listed in AURORA UNIVERSITIES NETWORK yielded more than 1 million documents relevant to keywords in the fields of the 17 SDGs. However, the author is interested in documents in which the phrase “sustainable development goal*” is mentioned in any place in the study. When the phrase “sustainable development goal*” was added to the overall search query of the 1 million documents and the time was limited from 2015 to 2019, the result was 18,696 publications in peer-reviewed journals. This number represents approximately 2.0% of the total number of publications with keywords listed in AURORA website and related to the 17 SDGs.

### Bibliometric indicators

The information retrieved from the Scopus database included: annual number of publications, active countries, active journals, active institutions, and citation information. Data in Scopus was exported to Excel software for tabulation or mapping. The number of publications on each SDG goal was analyzed using the search query in the AURORA UNIVERSITIES NETWOR for that goal after adding the phrase “sustainable development goal*” in all fields. The same method was applied to find out the research output for each target in SDG 3.

### Visualization

Data obtained for SDG 3 was exported to VOSviewer program for mapping purposes [22]. Mapping was made for (1) the most frequently encountered terms in titles/abstracts of the retrieved documents and (2) for countries with a minimum contribution of 20 documents to visualize international research collaboration in SDG 3.

## Results

### Volume and annual growth of publications

**Fig. 1:**
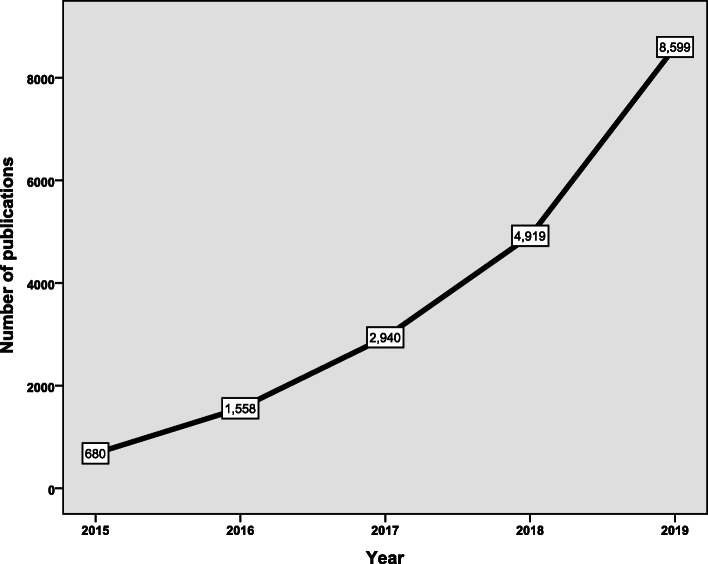
Annual growth of SDGs-related publications (2015–2019)

The search query found 18,696 documents. The majority of documents were research articles (*n* = 14,651; 78.4%) followed by review articles (*n* = 2367; 12.7%). The annual growth of publications showed steep growth (Fig. 1). The number of publications in 2019 was approximately 1.7 times the number of publications in 2018. Of the retrieved publications, 10,617 (56.8%) declared receiving funding for the study. The most active funding sponsor was Bill and Melinda Gates Foundation BMGF (*n* = 459; 2.5%).

### SDG goals

The retrieved documents were analyzed for the 17 SDGs (Table 1). The most researched goal was SDG 17 (partnership) followed by SDG 13 (climate action), 12 (responsible consumption and production), 15 (life on land), SDG 3 (good health and well-being), and SDG 1 (no poverty). The least researched goal was SDG 7 (affordable and clean energy) followed by SDG 10 (reduced inequality), 5 (gender equality), and 16 (peace, justice, and strong institutions). Documents on SDG 7, 3 (good health and well-being), and 13 received the highest number of citations per document while documents on SDG 4 (quality education) and 9 (industry, innovation, and infrastructure) received the least number of citations per document. For all SDG goals, the USA and the UK were among the top five active countries. China was among the top five active countries for SDGs 6, 7, 11, and 15.

**Table 1:** Number of publications on each SDG (2015–2019)

SDG Goal	Number of documents	% ***N*** = 18,696	Number of citations per document	Top five countries
1: No Poverty	1401	7.5	9.5	US, UK, South Africa, Australia, Canada
2: Zero Hunger	655	3.5	11.6	US, UK, Australia, Netherlands, Germany
3: Good Health and Well-being	1477	7.9	14.3	US, UK, Switzerland, Australia, Canada
4: Quality Education	685	3.7	5.1	UK, US, Australia, Germany, Canada
5: Gender Equality	403	2.2	11.0	US, UK, South Africa, Switzerland, Canada
6: Clean Water and Sanitation	781	4.2	10.5	US, UK, China, Netherland, Germany
7: Affordable and Clean Energy	203	1.1	14.6	Australia, US, Germany, China, UK
8: Decent Work and Economic Growth	493	2.6	9.4	US, UK, Australia, Germany, South Africa
9: Industry, Innovation and Infrastructure	550	2.9	9.7	US, UK, Australia, Germany, South Africa
10: Reduced Inequality	257	1.4	6.7	US, UK, Australia, South Africa, Canada
11: Sustainable Cities and Communities	1316	7.0	10.4	UK, US, China, Australia, Germany
12: Responsible Consumption and Production	1764	9.4	13.4	US, UK, Germany, Australia, Italy
13: Climate Action	2699	14.4	13.5	US, UK, Australia, Germany, Netherlands
14: Life Below Water	652	3.5	10.0	US, UK, Australia, Canada, Germany
15: Life on Land	1499	8.0	12.3	US, UK, Germany, Australia, China
16: Peace and Justice Strong Institutions	486	2.6	9.4	US, UK, South Africa, Australia, Canada
17: Partnerships to achieve the Goal	3073	16.4	10.2	US, UK, Australia, Switzerland, Germany
**Total**^**a**^	**18,375**	**98.4**		**US, UK, Australia, Germany, China**

^a^The total was less than the overall number (*n* = 18,696) either because of the presence of limited number of false positive results or because the keywords used to retrieve the documents on each SDG were not 100% accurate. The difference, which is approximately 1.5%, will not affect the overall analysis

### Annual growth of the top five SDG goals

**Fig. 2:**
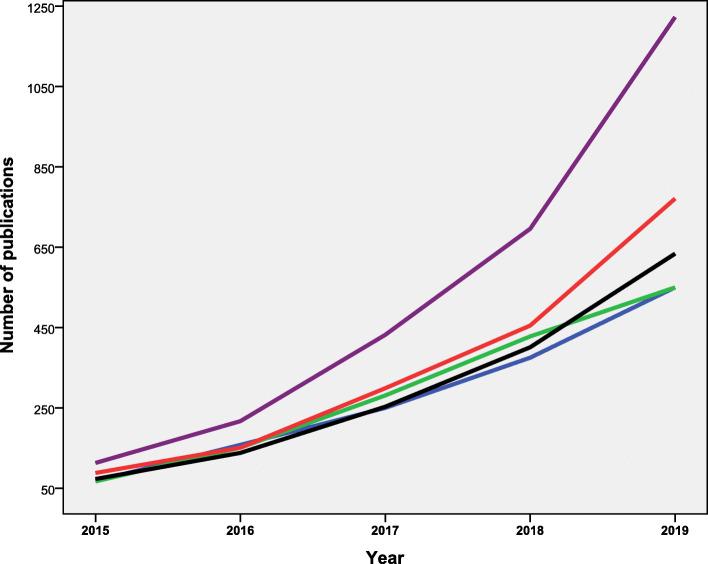
Annual growth of publication in top five SDGs (excluding SDG 17). Coding of the lines: Purple = SDG 13 (climate action); Red = SDG 12 (responsible consumption and production); Black = SDG 3 (good health and well-being); Green = SDG 15 (life on land); Blue = SDG 1 (no poverty)

The annual growth of the five SDGs with the highest number of publications (excluding SDG 17, which represents partnership) is depicted in Fig. 2. The climate action (SDG 13) showed the steepest growth with time while the growth of the remaining SDGs (Good health, Responsible consumption and production, No poverty, and Life on land) showed similar annual growth pattern.

### Subject areas of the retrieved documents

The retrieved documents were published in journals that belonged to different subject areas. Approximately 21% of the retrieved documents were published in journals within the subject area of social sciences. Subject areas of medicine and environmental sciences ranked second with 19% for each. Agriculture/biological sciences and energy subject areas ranked third and fourth with 8 and 7% respectively.

### Geographic distribution of the retrieved documents

**Fig. 3:**
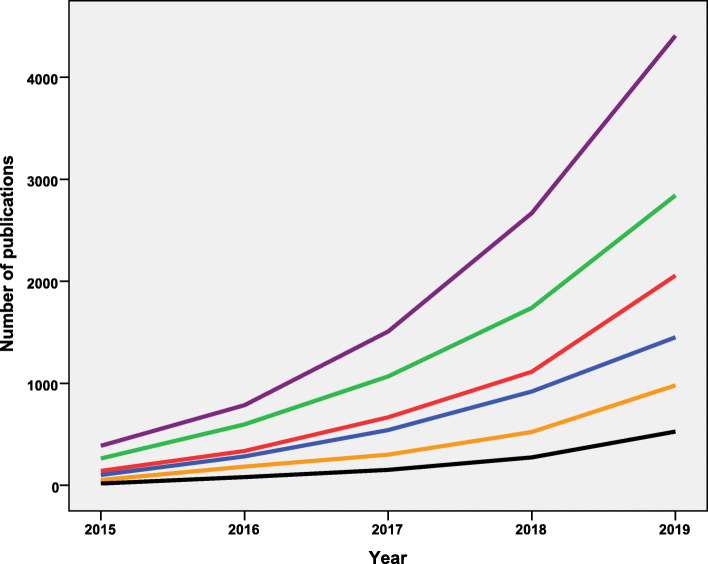
Annual growth of SDGs-related publications from different world regions. Coding of the lines: Purple = the European region; Green = the region of the Americas; Red = the Western Pacific region; Blue = the African region; Orange = the South-East Asian region; Black = the Eastern Mediterranean region

Table 2 lists the contribution of each WHO region to the retrieved documents. The European region (*n* = 9756; 52.2%) had the highest research contribution followed by the region of the Americas (*n* = 6512; 34.8%) and the Western Pacific region (*n* = 4315; 23.1%). The African region (*n* = 3299; 17.6%) made higher contribution than either the Eastern Mediterranean region (*n* = 1052; 5.6%) or the South-Eastern Asian region (*n* = 2041; 10.9%). Figure 3 shows the annual growth from different world regions. The annual growth in the six world regions showed a similar pattern with a leading role for the European region.

**Table 2:** Number of publications on 17 SDGs across different world regions

Region	African region ***N*** = 3299	Americas ***N*** = 6512	European ***N*** = 9756	South-East Asian ***N*** = 2041	Eastern Med. ***N*** = 1052	Western Pacific ***N*** = 4315
**Total number of countries**	**47**	**35**	**54**	**11**	**21**	**27**
**SDG number**	n (%)	n (%)	n (%)	n (%)	n (%)	n (%)
**SDG 1**	**401 (12.2)**	**538 (8.3)**	**700 (7.2)**	**204 (10.0)**	**64 (6.1)**	**290 (6.7)**
**SDG 2**	**148 (4.5)**	**253 (3.9)**	**362 (3.7)**	**96 (4.7)**	**29 (2.8)**	**138 (3.2)**
**SDG 3**	**468 (14.2)**	**707 (10.9)**	**693 (7.1)**	**249 (12.2)**	**143 (13.6)**	**313 (7.3)**
**SDG 4**	**116 (3.5)**	**199 (3.1)**	**371 (3.8)**	**69 (3.4)**	**38 (3.6)**	**122 (2.8)**
**SDG 5**	**117 (3.5)**	**175 (2.7)**	**186 (1.9)**	**60 (2.9)**	**23 (2.2)**	**57 (1.3)**
**SDG 6**	**143 (4.3)**	**292 (4.5)**	**397 (4.1)**	**93 (4.6)**	**48 (4.6)**	**196 (4.5)**
**SDG 7**	**15 (0.5)**	**43 (0.7)**	**103 (1.1)**	**16 (0.8)**	**20 (1.9)**	**72 (1.7)**
**SDG 8**	**76 (2.3)**	**181 (2.8)**	**269 (2.8)**	**46 (2.3)**	**14 (1.3)**	**127 (2.9)**
**SDG 9**	**119 (3.6)**	**199 (3.1)**	**294 (3.0)**	**33 (1.6)**	**43 (4.1)**	**146 (3.4)**
**SDG 10**	**44 (1.3)**	**87 (1.3)**	**136 (1.4)**	**20 (1.0)**	**9 (0.9)**	**58 (1.3)**
**SDG 11**	**117 (3.5)**	**338 (5.2)**	**711 (7.3)**	**149 (7.3)**	**72 (6.8)**	**408 (9.5)**
**SDG 12**	**162 (4.9)**	**535 (8.2)**	**1068 (10.9)**	**134 (6.6)**	**80 (7.6)**	**448 (10.4)**
**SDG 13**	**294 (8.9)**	**874 (13.4)**	**1630 (16.7)**	**247 (12.1)**	**118 (11.2)**	**798 (18.5)**
**SDG 14**	**58 (1.8)**	**254 (3.9)**	**376 (3.9)**	**80 (3.9)**	**19 (1.8)**	**227 (5.3)**
**SDG 15**	**164 (5.0)**	**503 (7.7)**	**861 (8.8)**	**146 (7.2)**	**106 (10.1)**	**439 (10.2)**
**SDG 16**	**106 (3.2)**	**191 (2.9)**	**243 (2.5)**	**60 (2.9)**	**24 (2.3)**	**83 (1.9)**
**SDG 17**	**538 (16.3)**	**1139 (17.5)**	**1704 (17.5)**	**320 (15.7)**	**181 (17.2)**	**646 (14.9)**
**TOTAL**^**a**^	**3086 (93.5%)**	**6508 (99.9%)**	**10,104 (103.0%)**	**2022 (99.1)**	**1031 (98%)**	**4567 (105.1%)**
**Top three researched SDGs (excluding SDG 17)**	**Health** **Poverty** **Climate action**	**Climate action** **Health** **Poverty**	**Climate action** **Responsible consumption and production** **Life on land**	**Health** **Climate action** **Poverty**	**Health** **Climate action** **Life on land**	**Climate action** **Responsible consumption and production** **Life on land**
**Least three researched SDGs**	**Clean energy** **Inequalities** **Life below water**	**Clean energy** **Inequality** **Gender equality**	**Clean energy** **Inequality** **Gender equality**	**Clean energy** **Gender equality** **Industry innovation**	**Inequality** **Life below water** **Gender equality**	**Gender equality** **Inequality** **Clean energy**

^a^The total for each world region was slightly lower or higher than 100% because of the presence of limited number of false positive results or because the keywords used to retrieve the documents on each SDG were not 100% accurate

### SDGs across different world regions

Table 2 also shows extensive analysis of the number of publications for each of the 17 SDGs for each world region. For all world regions, except for the Western Pacific region, the number of publications regarding partnership across all SDGs was the highest. However, after exclusion of SDG 17, the SDG 3 (good health and well-being) was the top researched SDG for the African region, the Eastern Mediterranean regions, and the South-Eastern Asian region. However, for the region of the Americas, European region, and the Western Pacific region, the SDG 13 (climate action) was the most researched. SDG 7 (affordable and clean energy) was the least researched in the African region, the region of the Americas, the European region, and the South-East Asian region. In the Eastern Mediterranean region, SDG 10 (reduced inequality) was the least researched while in the Western Pacific region, SDG 5 (gender inequality) was the least researched.

### Top ten active journals

The *Sustainability*, a multidisciplinary journal ranked first (*n* = 1008; 5.4%) in the number of SDG-linked publications followed by *PloS One* (*n* = 373; 2.0%) and *Journal of Cleaner Production* (*n* = 347; 1.9%). Nine of the top 10 active journals were based in Europe and one was based in the USA (Table 3).

**Table 3:** Top ten active journals in publishing SDGs-related research (2015–2019)

Rank	Journal	Frequency (%) ***N*** = 18,696	Rank^**a**^	Country	Scope of the journal^**b**^
1	*Sustainability Switzerland*	1008 (5.4)	Q2	Switzerland	Energy, Renewable Energy, Sustainability and Environmental Science
2	*Plos One*	373 (2.0)	Q1	USA	Agricultural and Biological Sciences (miscellaneous),
3	*Journal Of Cleaner Production*	347 (1.9)	Q1	Netherlands	Business, Management and Accounting; Strategy and Management Energy, Renewable Energy, Sustainability and the Environment
4	*Lancet*	269 (1.4)	Q1	UK	Medicine (miscellaneous)
5	*Science Of The Total Environment*	223 (1.2)	Q1	Netherlands	Environmental Science
6	*International Journal Of Environmental Research And Public Health*	198 (1.1)	Q2	Switzerland	Medicine, Public Health, Environmental and Occupational Health
7	*Lancet Global Health*	169 (0.9)	Q!	UK	Medicine
8	*BMJ Global Health*	164 (0.9)	Q!	UK	Medicine, Health Policy, Global health, Public health, Environmental and Occupational Health
9	*BMC Public Health*	134 (0.7)	Q1	UK	Medicine, Public Health, Environmental and Occupational Health
10	*Water Switzerland*	134 (0.7)	Q1	Switzerland	Agricultural and Biological Sciences, Aquatic Science,

^a^Rank is obtained from Scimajo Journal Ranking. Q1 represents the highest rank and Q4 represents the lowest rank

^b^Information on the scope of the journals was obtained from Scimajo Journal and Country Rank

### Top ten active institutions

Analysis of active institutions based on author affiliation indicated that the WHO was the most active institution in publishing SDG-linked documents (*n* = 581; 1.3%) followed by *London School of Hygiene & Tropical Medicine* (*n* = 492; 2.7%), and *Harvard University* (*n* = 483; 2.3%) (Table 4). The top ten active institutions included nine academic institutions in addition to the WHO.

**Table 4:** Top ten active institutions in publishing SDGs-related research (2015–2019)

Rank^**a**^	Institutions	Frequency (%) ***N*** = 18,696	Country
1	*Organisation Mondiale de la Santé*	581 (3.1)	United Nations
2	*London School of Hygiene & Tropical Medicine*	492 (2.6)	UK
3	*Harvard University*	483 (2.6)	USA
4	*Johns Hopkins University*	375 (2.0)	USA
6	*University College London*	346 (1.9)	UK
6	*University of Oxford*	339 (1.8)	UK
7	*Wageningen University & Research*	286 (1.5)	The Netherlands
8	*University of Washington, Seattle*	282 (1.5)	USA
9	*University of Melbourne*	259 (1.4)	Australia
10	*University of Cape Town*	254 (1.4)	South Africa

^a^Equally active institutions were given the same rank

### SDG 3 (good health and well-being)

The number of SDG 3-related publications was 1477. The distribution of these publications on the health targets was shown in Table 5. The most researched targets were targets 7 (sexual and reproductive health services) and 8 (universal health coverage) while the least researched targets were 5 (substance use disorders) and 9 (death from hazardous materials).

**Table 5:** Number of publications for each target in SDG 3

SDG 3 Target	Description	Number of publications	% ***N*** = 1477
**Target 1**	By 2030, reduce the global maternal mortality ratio to less than 70 per 100,000 live births.	155	10.5
**Target 2**	By 2030, end preventable deaths of newborns and children under 5 years of age, with all countries aiming to reduce neonatal mortality to at least as low as 12 per 1000 live births and under-5 mortality to at least as low as 25 per 1000 live births.	68	4.6
**Target 3**	By 2030, end the epidemics of AIDS, tuberculosis, malaria and neglected tropical diseases and combat hepatitis, water-borne diseases and other communicable diseases.	127	8.6
**Target 4**	By 2030, reduce by one third premature mortality from non-communicable diseases through prevention and treatment and promote mental health and well-being.	148	10.0
**Target 5**	Strengthen the prevention and treatment of substance abuse, including narcotic drug abuse and harmful use of alcohol.	6	0.4
**Target 6**	By 2020, halve the number of global deaths and injuries from road traffic accidents.	46	3.1
**Target 7**	By 2030, ensure universal access to sexual and reproductive health-care services, including for family planning, information and education, and the integration of reproductive health into national strategies and programmes.	246	16.7
**Target 8**	Achieve universal health coverage, including financial risk protection, access to quality essential health-care services and access to safe, effective, quality and affordable essential medicines and vaccines for all.	607	41.1
**Target 9**	By 2030, substantially reduce the number of deaths and illnesses from hazardous chemicals and air, water and soil pollution and contamination.	10	0.7
**3a**	Strengthen the implementation of the WHO Framework Convention on Tobacco Control in all countries, as appropriate.	49	3.3
**3b**	Support the research and development of vaccines and medicines for the communicable and non-communicable diseases that primarily affect developing countries, provide access to affordable essential medicines and vaccines, in accordance with the Doha Declaration on the TRIPS Agreement and Public Health, which affirms the right of developing countries to use to the full the provisions in the Agreement on Trade-Related Aspects of Intellectual Property Rights regarding flexibilities to protect public health, and, in particular, provide access to medicines for all.	41	2.8
**3c**	Substantially increase health financing and the recruitment, development, training and retention of the health workforce in developing countries, especially in least developed countries and small island developing States.	160	10.8
**3d**	Strengthen the capacity of all countries, in particular developing countries, for early warning, risk reduction and management of national and global health risks.	18	1.2

### Mapping SDG 3

**Fig. 4:**
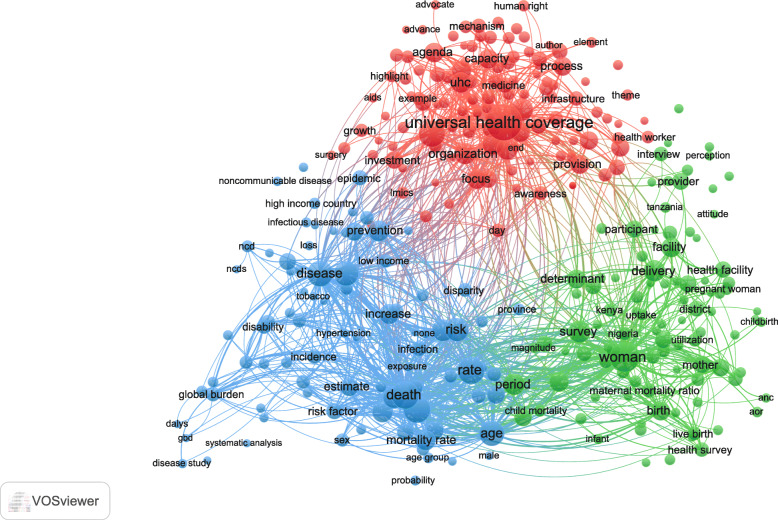
Network visualization map of SDGs-related publications on SDG 3. Three colored clusters represent three major research themes

Network visualization of the terms in titles and abstracts of SDG 3 related publications showed three distinct clusters representing three major research themes (Fig. 4): universal health coverage (red color), women/maternal health (green color), and the global burden of diseases (blue color).

### International research collaboration in SDG 3-linked documents

**Fig. 5:**
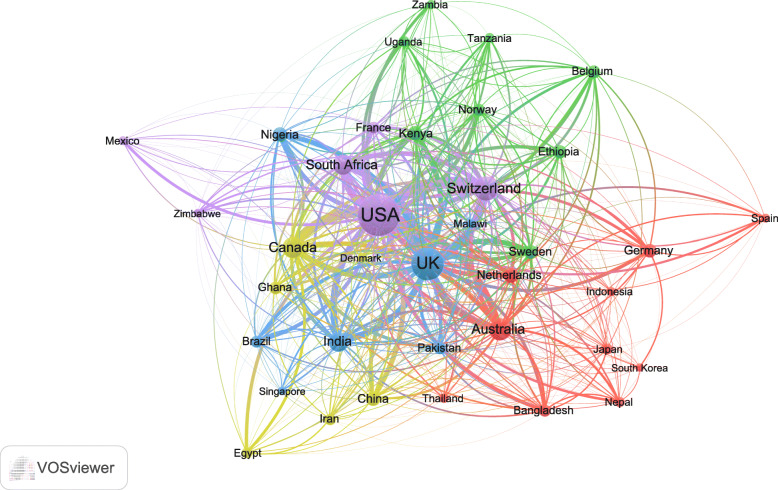
Network visualization map of international research collaboration among countries with a minimum of 20 SDGs-related publications. Countries in the center with many connections had the highest research collaboration while countries at the edge of the map had the least research collaboration

Network visualization of countries with a minimum contribution of 20 documents for SDG 3 – linked publications (Fig. 5). The map was characterized by the presence of countries from poor regions such as Africa, the Eastern Mediterranean, and South-East Asian regions far away from the center and with limited connections with countries in the center of the map indicative of poor international research collaboration. Countries in the center of the map with the largest node size (the USA and the UK) had the largest research connections with other countries while countries such as Egypt, Zambia, and Nepal located at the margin of the map with small node sizes had the least international research collaborations in SDGs-related research field.

## Discussion

The current study was undertaken to assess and analyze scientific publications in which the phrase “sustainable development goal*” was mentioned in any part of the article assuming that such publications represent research related to one or more aspects of SDGs. The current study showed a steep increase in the number of SDGs-related publications with time reflecting an increasing interest of researchers in topics that are related to SDGs. It also reflects national and international support and commitment to achieve the SDGs. Research volume on SDGs of poverty (SDG 1), health (SDG 3), responsible consumption and production (SDG 12), climate action (SDG 12), sustainable cities and communities (SDG 11), and life on land (SDG 15) dominated the overall retrieved literature. A recent qualitative study using snowball approach for experts on SDGs research indicated that SDGs 11, 12, 13 and 15 were among the most researched SDGs while SDGs 8 and 14 were among the least researched ones which is in agreement of the findings presented in the current study [23]. Another recent study that mapped the Australian research on SDGs found that SDGs number 11, 12, 13, and 15 were among the most researched SDGs while SDGs 14 and 5 were the least researched [24]. It seems that there are regional differences regarding research interest in various SDGs. For example, SDG experts in Northern America and the European region pointed to SDGs 4, 11, 12, and 13 as the most preferred ones while specialists in the African region indicated that SDGs 13, 1, 2, 5, 6, and 15 were most important [23].

The current study showed that most researched SDG goals were those related to health, environment, and science. However, when overall literature was considered, the bulk of the retrieved documents were in social sciences, followed by medicine and environmental science subject areas. This was not surprising given that at least half of the SDGs are related to social issues such as poverty, hunger, education, inequality, gender, and peace. In fact, social and health targets in the SDGs represent global problems that need tremendous research and solution [25–34]. Environmental issues such as climate change and global warming are also a major global challenge to life and economy for planet earth [34, 35].

In the current study, most active journals were in the Q1 rank suggesting that the SDG – related papers are of interest to journal editors. This also suggests that research content and quality of SDGs-related publications are high and beneficial for policymakers. None of the active journals was based outside Europe or North America. This might be one possible reason for the large contribution of European and North American countries to SDGs-related literature compared to other countries that lack scientific journals indexed in Scopus. The current study indicated the *Sustainability* journal ranked first followed distantly by the *PloS One* journal. The finding regarding the list of top 10 active journals is in agreement with the findings of a recent paper on sustainable development goals in which the author used the title-abstract-key to search for SDG-related documents [19]. This finding confirms that the methodology adopted in the current study was valid and the difference between the two approaches is in the number of the retrieved documents while the findings were relatively close. In the study by Christine Meschede [19], a total of 4600 documents were retrieved and analyzed compared with approximately 18,700 documents retrieved and analyzed in the current study. However, the findings regarding the active journal list and other bibliometric indicators were close.

Universities and academics in various countries are in a position to interact and have a leading role through research collaboration on various SDGs. The finding that nine of the top 10 active institutions were academic institutions reflects the growing role of academics in the implementation and research on SDGs. Researchers and academic staff must teach and train today’s students, tomorrow’s decision, and policymakers, to think on genuine approaches to solve global serious and complex societal and economic problems [36]. Academic institutions need to integrate SDGs in research, teaching, and student training to become leaders of change at the national and global levels.

The current study showed that countries in the African region, the Eastern Mediterranean region, and the South-East Asian region had the least contribution to SDGs-related research publications. The online database for Sustainable Development Report 2020, showed that the achievements of most countries in these regions were coded red or orange suggesting major or significant challenges in goal achievements across the 17 SDGs. In contrast, most countries with green or yellow coding for the 17 SDGs were based in the European region, which could be attributed, in part, to the outstanding research output from this region. The current study indicated that the US and the UK were in the frontline regarding research activity across the 17 SDGs. However, the Sustainable Development Report 2020 indicated that the US is facing significant and even major challenges in many of the 17 SDGs. This might suggest that national research output in SDGs does not significantly correlate with the SDG Index score or goal achievement. Research activity is one important factor in creating knowledge and innovation that will lead to sustainable growth and development. However, politics is also a major key player in this regard and might facilitate or hinder goal achievement.

The current study showed that SDG17, which refers to partnership for the goals, was the most researched item as an overall and for each world region. The SDGs included 15 targets and has the largest number of keywords in its search query as presented in the Aurora Universities Network web site. The SDG 17 demands partnerships between governments, the private sector and civil society. The type of research publications that have been analyzed in the current study is the one in which SDG phrase was mentioned and we expect that such research focuses on policies and agendas that require governmental and private sector partnership. If all research on SDGs was included, i.e. the 1 million documents, we expect that SDG 17 will be one of the top researched goals but not number one. Similar findings were presented in a recently published article [19].

The current study indicated that SDG 3, which was one of the top researched goal across different world regions, was characterized by inadequate international research collaboration. An important aspect of SDGs is the global approach and partnership. The current study showed that the extent and strength of research collaboration in SDGs-related research vary based on the geographical region. European and American researchers have relatively strong collaboration while research collaboration with researchers in Asian or African countries was poor. Policymakers in regions with low research output in SDGs need to encourage and support joint research with European and North American researchers. Such research collaboration is believed to attract more funding and better chances of publishing higher impact documents [37, 38].

Analysis of SDG 3 indicated that the prevention and treatment of substance use disorder was the least researched target. According to the WHO, some 31 million persons have drug use disorders at the global level with almost 11 million people injecting drugs. Substance use disorders have been associated with increased risk of HIV, tuberculosis, hepatitis and may even accelerate the spread of serious pandemics such as COVID-19 [5, 39–41]. The social stigma attached to drug users acts as a strong barrier to improve support services [42]. In several parts of the world, governments impose compulsory detention with beating and humiliation of people who use drugs in the name of treatment [43, 44]. Such human rights violations are not in accordance with SDG 3.5 and research on preventive and management policies of drug users need to be carried out in all parts of the world.

The BMGF was the most active in funding SDGs-related research. The BMGF is the largest private foundation in the world with the primary aims of enhancing healthcare and reducing extreme poverty in the world. At the USA level, the foundation aims to expand educational opportunities and access to information technology. Funding is a major driving force for publications, growth of scientific collaboration, and enhanced impact of scientific articles [45–49].

### Limitations

The present study has some limitations. First, our study did not include articles published in non-Scopus databases. This negatively affected the number of publications retrieved from countries/regions with local or regional journals that are not indexed in Scopus. Second, the current study focused on peer-reviewed literature and did not include grey literature such as governmental reports. Sometimes, grey literature included important information and assessment of the national progress in implementing SDGs. Third, all types of publications such as notes, letters, reviews, and editorials were included in the analysis. However, these types might not represent original research contribution. It is well known that *The Lancet* has many editorials and such documents might overestimate the role of the journal in publishing documents on SDGs. Fourth, the method adopted in the current study did not distinguish articles that focus on SDGs from those that only mention the term. The opposite is also true. A document might discuss a problem such as sanitation without mentioning the phrase SDGs. Therefore, this limitation should be taken into consideration when reading the current study. Despite all this, the current study provides a comprehensive picture of SDGs-related research productivity and subject areas of interest to worldwide researchers.

## Conclusion

Research, innovation, education, and funding are crucial elements to achieve the SDGs. These elements need to be investigated periodically to assess the involvement of academia and researchers in partnership toward achieving the global 2030 agendas. The results of the current study showed that SDGs-related literature published in peer-reviewed journals were growing rapidly with an obvious emphasis on SDG 3 and SDG 13. The majority of publications came from the European regions while the Eastern Mediterranean region had the least contribution. Good health and well-being (SDG 3) was the focus of researchers in low-income regions such as Africa, the Eastern Mediterranean, and the South-East Asia. Climate action (SDG 13) was the focus of researchers in high-income regions such as the Americas, Europe, and Western Pacific regions.

## Supplementary information

**Additional file 1.**

## Data Availability

All data presented in this manuscript are available on Scopus database using the search query listed in the methodology section.

## Abbreviations

GDP: Gross Domestic Product
SDG: Sustainable development goals
