# Supplementary material for: Bibliometric analysis of scientific publications on “sustainable development goals” with emphasis on “good health and well-being” goal (2015–2019)
Source: Global Health. 2020 Jul 28;16:68. doi: 10.1186/s12992-020-00602-2 (PMC7385333; doi:10.1186/s12992-020-00602-2)
Supplement: Supplementary file 1 — Additional file 1. [file 12992_2020_602_MOESM1_ESM.docx]

**Additional file 1**

**Search strategy for retrieving documents on SDG-linked publications from Scopus (2015 – 2019)**

**The number of documents retrieved from Scopus (2015 – 2019) on SDGs was obtained using the following search query**

**ALL("sustainable development goal*") = 18,696**

**Number of documents on each SDG goal for each world region was calculated using the above search query along with the list of all keywords for each SDG goal obtained from the AURORA UNIVERSITIES NETWOK**

**The number of documents on SDGs for each world region was calculated using the above search query along with the list of all countries in that region.**

**ALL("sustainable development goal*") AND COUNTRY AFFILIATION (list of all countries in the specified region)**
